# Synthesis and crystal structure of the cubane-like cluster [(Tp*)MoFe_3_S_3_(μ_3_-Cl)(PMe_3_)_3_](BPh_4_)

**DOI:** 10.1107/S2056989026005475

**Published:** 2026-06-05

**Authors:** Juan He, Xuan-Yi Chen, Jia Wei, Jie Han, Xu-Dong Chen, Gan Xu

**Affiliations:** a Jiangsu Collaborative Innovation Center of Biomedical Functional Materials School of Chemistry and Materials Science Nanjing Normal University,Nanjing 210023 Jiangsu People’s Republic of China; b Changzhou University, Wujin District Changzhou City Jiangsu Province 213164 People’s Republic of China; c School of Science and Technology Hong Kong Metropolitan University Hong Kong; Harvard University, USA

**Keywords:** Mo–Fe–S cluster, phosphine ligand, crystal structure, synthesis

## Abstract

The title compound, μ_3_-chlorido-tri-μ_3_-sulfido-tris­(tri­methyl­phosphine)[tris­(3,5-di­methyl­pyrazol-1-yl)hydro­borato]triiron(II)molybdenum(III) tetra­phenyl­borate, [MoFe_3_S_3_Cl(C_15_H_22_BN_6_)(C_9_H_27_P_3_)](C_24_H_20_B) or [(Tp*)MoFe_3_S_3_(μ_3_-Cl)(PMe_3_)_3_](BPh_4_) [Tp* = tris­(3,5-di­methyl­pyrazol-1-yl)hydro- borate(1-)], crystallizes as block-shaped crystals in space group *P*1. In this cluster, the Mo site is in a distorted octa­hedral coordination model, coordinating to three N atoms on the Tp* ligand and three μ_3_-bridging S atoms in the core. The Fe sites adopt a distorted tetra­hedral coordination geometry, each coordinating to two μ_3_-bridging S atoms, one μ_3_-bridging Cl atom, and the phospho­rus atom from a tri­methyl­phosphine ligand. This type of heterometallic and heteroleptic single cubane cluster represents a typical example within the Mo–Fe–S cluster family. It may possess unique advantages in the construction of high-nuclearity clusters, and could serve as a potentially controllable reaction inter­mediate for the synthesis of FeMo-co mimics. The residual electron density of disordered solvent mol­ecules in the void space could not be reasonably modeled, thus a solvent mask was applied. The solvent contribution is not included in the reported mol­ecular weight and density.

## Abstract 2

The title heterometallic single cubane cluster [(Tp*)MoFe_3_S_3_(μ_3_-Cl)(PMe_3_)_3_](BPh_4_) [Tp* = tris­(3,5-di­methyl­pyrazol-1-yl)hydro­borate(1–)] features distinct coordination geometries for the Mo and Fe sites and serves as a typical example in the Mo–Fe–S cluster family with potential applications for the construction of high-nuclearity clusters and synthesis of FeMo-co mimics.

## 1. Chemical context

The chemistry of Mo–Fe–S clusters has received extensive and sustained attention, mainly due to their significant structural and functional similarity to the iron–molybdenum cofactor (FeMo-co) of nitro­genase (Hoffman *et al.*, 2014 ▸; Burgess & Lowe, 1996 ▸; Burén *et al.*, 2020 ▸), which catalyzes di­nitro­gen reduction to ammonia under ambient conditions. Cubane-type Mo–Fe–S clusters act as vital structural and functional mimics of the nitro­genase active site, providing reliable clues for understanding biological nitro­gen fixation and promoting the development of artificial biomimetic models with tunable reactivity (Venkateswara Rao & Holm, 2004 ▸; Lee *et al.*, 2014 ▸; Lee & Holm, 2004 ▸). In such heterometallic systems, terminal ligands at iron centers dominantly regulate the electronic structure, local coordination environment and core reactivity (Palermo *et al.*, 1984 ▸; Pesavento *et al.*, 2007 ▸; Koutmos *et al.*, 2006 ▸). Phosphine ligands are among the most common terminal ligands in Mo–Fe–S cluster chemistry (Zhang *et al.*, 2002 ▸; Zhang & Holm, 2003 ▸; Berlinguette & Holm, 2006 ▸). Nevertheless, most reported phosphine-coordinated cubane iron–sulfur clusters contain inert μ_3_-sulfido bridges with low chemical activity, which greatly limits further structural modification and functional derivatization. In contrast, our group has developed a series of cubane-type Mo–Fe–S clusters bearing a labile μ_3_-chlorido ligand, which display superior reactivity and offer a new strategy for the rational design and controlled synthesis of high-performance iron–sulfur clusters (Xu *et al.*, 2018 ▸, 2019 ▸, 2025 ▸; He *et al.*, 2022 ▸; Qiu *et al.*, 2024 ▸; Zhang *et al.*, 2023 ▸; Xue *et al.*, 2021 ▸).
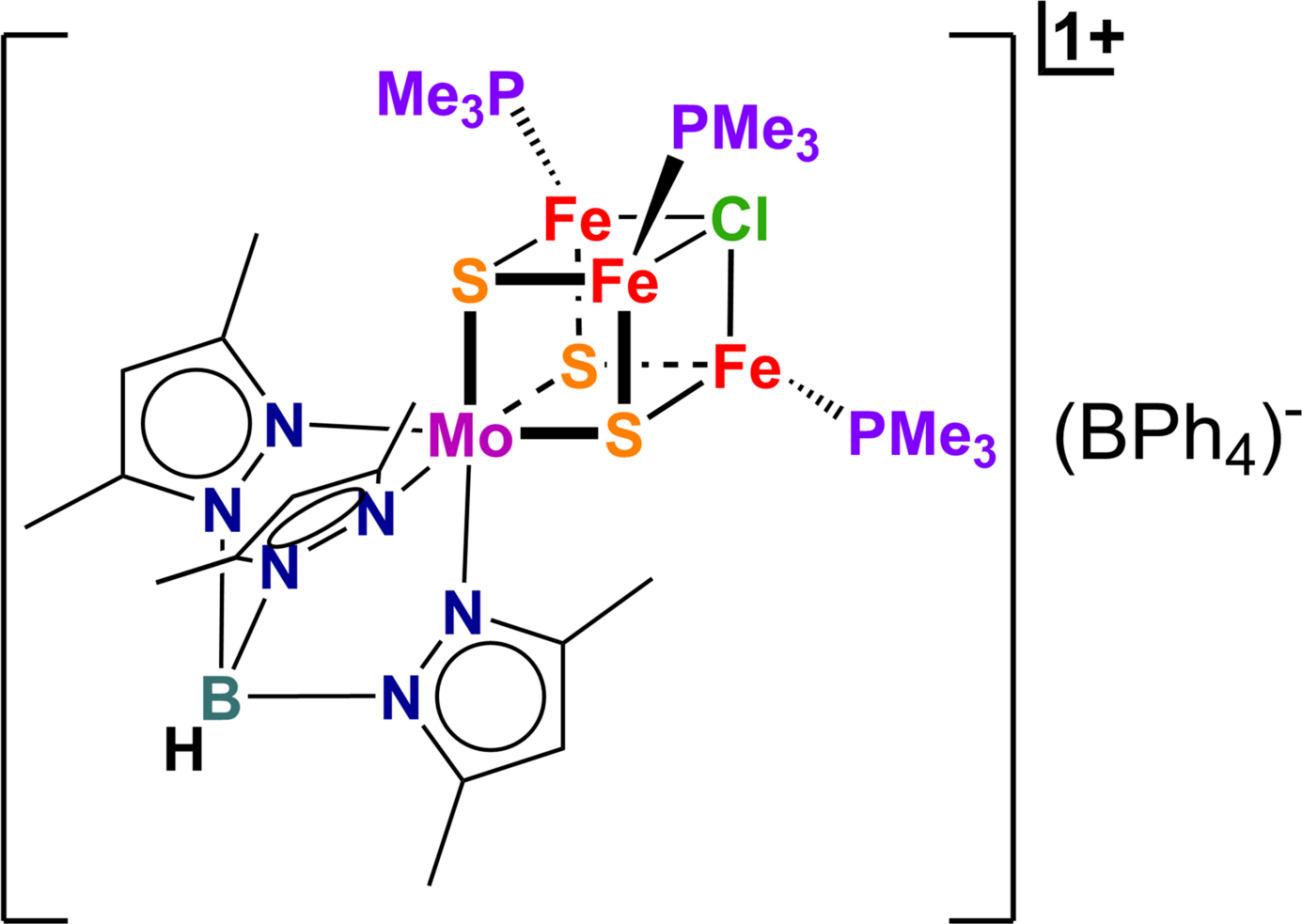


Previously, our group successfully synthesized the molybdenum–iron–sulfur precursor cluster (Et_4_N)_2_[(Tp*)MoFe_3_S_3_(μ_3_-Cl)Cl_3_] via a LEGO-like strategy (He *et al.*, 2022 ▸). This compound serves as an excellent precursor for the construction of phosphine-functionalized derivative clusters. On this basis, we systematically explored the regulation of terminal phosphine ligands and synthesized the new cubane cluster [(Tp*)MoFe_3_S_3_(μ_3_-Cl)(PMe_3_)_3_](BPh_4_) through ligand substitution. Its synthesis and single-crystal structural characterization enrich the structural diversity of phosphine-modified Mo–Fe–S cubane clusters and provide a clear structural model for nitro­genase mimic research. Importantly, the bridging μ_3_-chlorido ligand is structurally labile and readily substitutable (Xu *et al.*, 2018 ▸, 2019 ▸; He *et al.*, 2022 ▸), representing a key distinction from the inert bridging sulfido ligand widely reported in phosphine-ligated cubane clusters. This structural feature affords the cluster excellent derivatization capacity. It can act as a favorable reactive inter­mediate for subsequent core ligand metathesis, functional modification and high-nuclearity cluster assembly, and also provides solid experimental support for exploring the structure–activity relationships of Mo–Fe–S clusters.

## 2. Structural commentary

**Figure 1:**
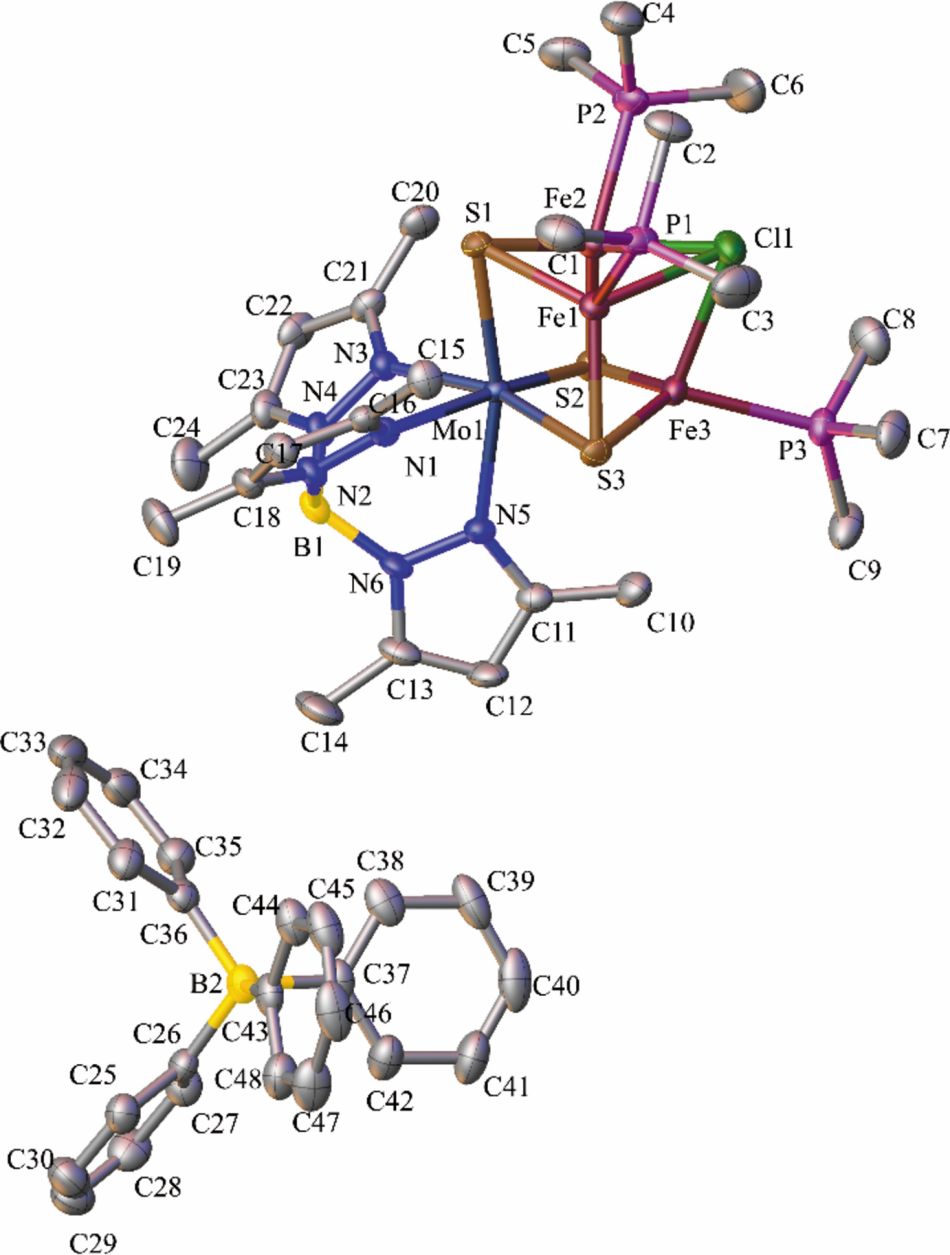
Structure of the title compound with the atom-numbering scheme. Displacement ellipsoids are drawn at the 50% probability level. Hydrogen atoms are omitted for clarity.

This title cluster crystallized as the BPh_4_^−^ salt in the triclinic crystal system, space group *P*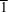. The different metal atoms exhibit distinct coordination environments in this cluster. The Mo site coordinates three N atoms of the Tp* ligand and three μ_3_-bridging S atoms in the core of the cluster, showing a distorted octa­hedral coordination sphere. Each Fe site coordinates to two μ_3_-bridging S atoms, one μ_3_-bridging Cl atom, and the phospho­rus atom from a tri­methyl­phosphine ligand. The cluster adopts quasi-threefold symmetry in the crystalline state, which is induced by the spatial confinement of crystal packing. In the core of the cluster, the Mo—S bond lengths range from 2.3928 (9) to 2.3986 (9) Å, with an average value of 2.395 (1) Å. The Mo⋯Fe distances are between 2.6955 (6) and 2.7080 (6) Å, averaging 2.702 (1) Å. The Fe⋯Fe distances fall in the range 2.5772 (8)–2.6085 (7) Å, with a mean value of 2.592 (1) Å. The Fe—S bond lengths range from 2.2387 (11) to 2.2573 (10) Å, with an average value of 2.249 (2) Å. The Fe—Cl bond lengths are in the range 2.4677 (11) to 2.4974 (11) Å, with an average value of 2.481 (2) Å. The Fe—P bond lengths are between 2.3930 (11) and 2.4019 (12) Å, with an average value of 2.399 (1) Å. The Fe—Cl—Fe angles range from 62.37 (3) to 63.64 (3)° with an average of 62.97 (3)°. The structure of the cluster [(Tp*)MoFe_3_S_3_(μ_3_-Cl)(PMe_3_)_3_](BPh_4_) is shown in Fig. 1 ▸ and some selected geometric parameters are listed in Table 1 ▸.

## 3. Supra­molecular features

**Figure 2:**
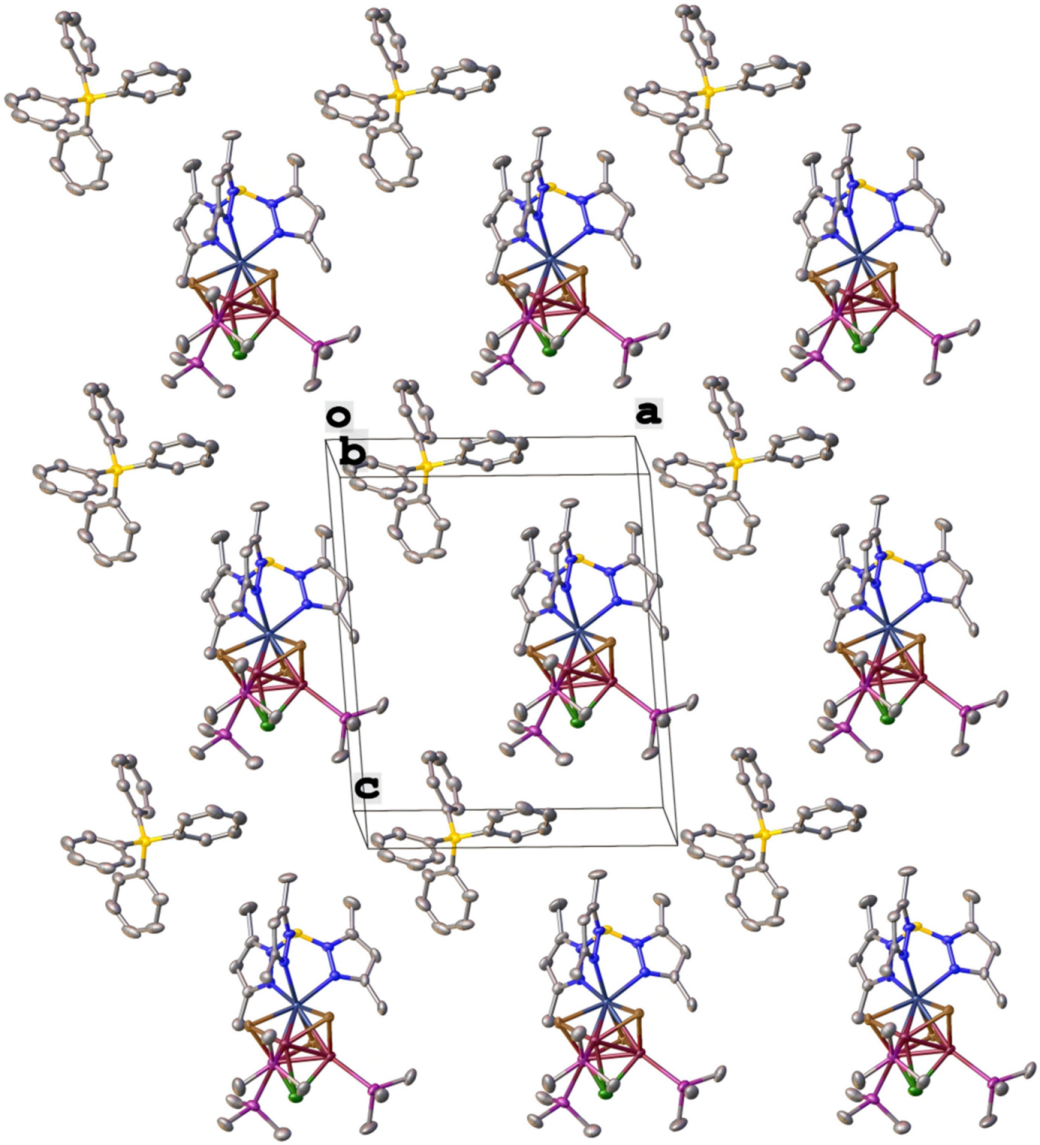
Crystal packing of the title compound. Hydrogen atoms are omitted for clarity.

**Figure 3:**
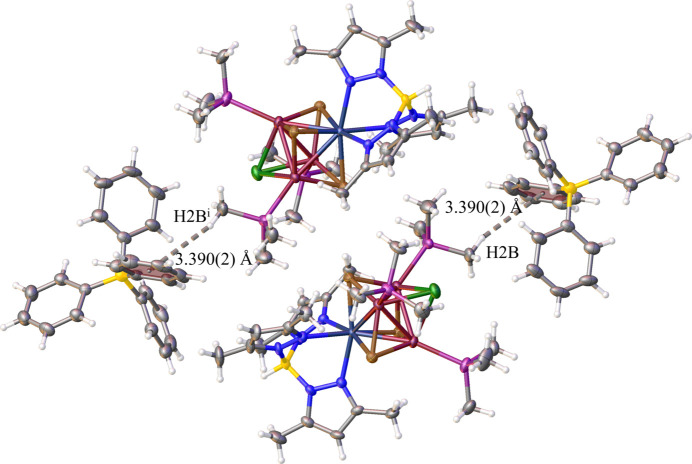
C—H⋯π inter­actions in the title compound (distances in Å); symmetry code: (i) 1 − *x*, 1 − *y*, 1 − *z*.

In the crystal, the title cluster exhibits a layered stacking arrangement along the *b*-axis direction. The cationic cluster units and BPh_4_^−^ anions are arranged in a parallel mode throughout the crystal. Electrostatic inter­actions dominate the supra­molecular assembly of the solid-state structure (Fig. 2 ▸). The crystal packing is further consolidated by weak inter­molecular inter­actions. Notably, a weak C—H⋯π inter­action is identified between the methyl C—H group of the tri­methyl­phosphine ligand and a benzene ring of the tetra­phenyl­borate anion, with an H⋯π distance of 3.39 Å (Huang *et al.*, 2016 ▸; Goswami *et al.*, 2021 ▸), symmetry code: (1 − *x*, 1 − *y*, 1 − *z*) (Fig. 3 ▸).

## 4. Database survey

Heteroleptic cubane-type *M*–Fe–S–Cl clusters (*M* = Mo, W) are rare. Only a limited number of cubane-type *M*–Fe–S–Cl clusters with diverse terminal ligands have been documented (Xu *et al.*, 2018 ▸, 2023 ▸, 2025 ▸; He *et al.*, 2022 ▸; Le *et al.*, 2021 ▸). Only two examples bearing phosphine ligands have been reported to date (Xu *et al.*, 2023 ▸, 2025 ▸).

A search of the Cambridge Structural Database with WebCSD (updated to February 2026; Groom *et al.*, 2016 ▸) revealed five examples of heteroleptic cubane-type *M*–Fe–S–Cl clusters (*M* = Mo, W), *viz*. (Et_4_N)_2_[(Tp*)WFe_3_S_3_(μ_3_-Cl)Cl_3_] (NIDZOS; Xu *et al.*, 2018 ▸); [(Tp*)WFe_3_S_3_(μ_3_-Cl)(PEt_3_)_3_](BPh_4_) (TOGBUQ; Xu *et al.*, 2023 ▸), [(Tp*)MoFe_3_S_3_(μ_3_-Cl)(PEt_3_)_3_](BPh_4_) (BACZUF; Xu *et al.*, 2025 ▸); (Et_4_N)_2_[(Tp*)MoFe_3_S_3_(μ_3_-Cl)Cl_3_] and [(Tp*)WFe_3_S_3_(μ_3_-Cl)(BAC)_3_](BPh_4_) (XATZOL01 and XASGEH01; Le *et al.*, 2021 ▸).

## 5. Synthesis and crystallization

**Figure 4:**
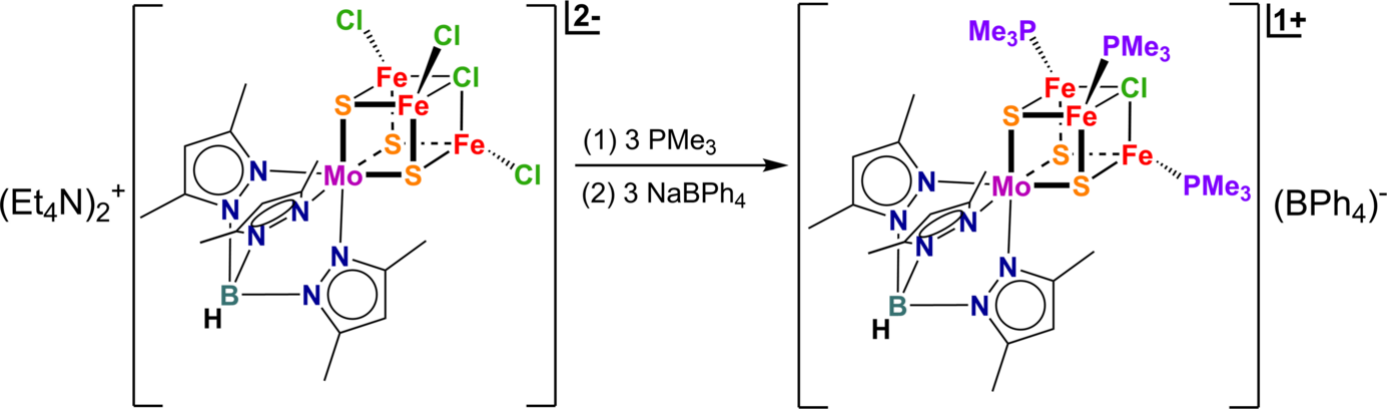
Synthesis of [(Tp*)MoFe_3_S_3_(μ_3_-Cl)(PMe_3_)_3_](BPh_4_).

All manipulations were conducted on standard Schlenk lines or in a glovebox under an atmosphere of dry nitro­gen. All glassware was subjected to a drying process in an oven maintained at a temperature of 403 K for a period exceeding three hours. Diethyl ether and tetra­hydro­furan were refluxed over sodium metal and benzo­phenone until completely dry, and then distilled under a dry nitro­gen atmosphere. All solvents were stored in a glovebox over activated mol­ecular sieves (3 Å). As shown in Fig. 4 ▸, the cluster compound (Et_4_N)_2_[(Tp*)MoFe_3_S_3_(μ_3_-Cl)Cl_3_] (52.9 mg, 0.05 mmol) was dispersed in 5 mL of THF. Then, 150 µL of tri­methyl­phosphine solution (1 *M* in THF) were added, followed by the addition of sodium tetra­phenyl­borate (51.3 mg, 0.05 mmol) dissolved in 2 mL of THF. Upon stirring at room temperature for 6 h, the reaction mixture changed color from blue to purple–red. The resulting mixture was filtered through celite, and the filtrate was subjected to diethyl ether vapor diffusion at room temperature to afford black block-shaped crystals (49.6 mg, 80%).^1^H NMR (400 MHz, CD_3_CN, δ, ppm): 7.28 (*s*, 8H, CH), 7.00 (*s*, 8H, CH), 6.85 (*s*, 4H, CH), 2.09 (*s*, 2H, CH), 1.53 (*s*, 3H, CH_3_), 1.32 (*s*, 9H, CH_3_). Other proton signals could not be located because of paramagnetic broadening (Scott & Agapie, 2022 ▸; Scott *et al.*, 2025 ▸; McSkimming & Suess, 2021 ▸). Elemental analysis: calculated for C_48_H_69_B_2_ClFe_3_MoN_6_P_3_S_3_: C, 46.50; H, 5.61; N, 6.78. Found: C, 46.12; H, 5.35; N, 6.56.

## 6. Refinement

Crystal data, data collection, and structure refinement details are summarized in Table 2 ▸. All hydrogen atoms were placed in idealized geometric positions and refined using a riding model. The residual electron density arising from disordered solvent mol­ecules within the crystal voids could not be satisfactorily modelled. Therefore, the solvent mask procedure implemented in *OLEX2* was employed to account for the disordered solvent contribution during the final refinement. A total of 58 electrons in a volume of 292 Å^3^ were counted by the solvent mask and removed per unit cell. This accounts for about 1.5 solvent mol­ecules (probably THF) per unit cell.

## Supplementary Material

Crystal structure: contains datablock(s) I. DOI: 10.1107/S2056989026005475/oi2038sup1.cif

Supporting information file. DOI: 10.1107/S2056989026005475/oi2038Isup3.mol

Structure factors: contains datablock(s) I. DOI: 10.1107/S2056989026005475/oi2038Isup5.hkl

CCDC reference: 2549922

Additional supporting information:  crystallographic information; 3D view; checkCIF report

## Figures and Tables

**Table 1:** Selected geometric parameters (Å, °)

Mo1—Fe1	2.7080 (6)	Fe1—S3	2.2387 (11)
Mo1—Fe2	2.7028 (6)	Fe1—Cl1	2.4678 (11)
Mo1—Fe3	2.6955 (6)	Fe1—P1	2.4006 (11)
Mo1—S1	2.3944 (9)	Fe2—Fe3	2.5772 (8)
Mo1—S2	2.3928 (9)	Fe2—S1	2.2470 (10)
Mo1—S3	2.3986 (9)	Fe2—S2	2.2536 (10)
Mo1—N1	2.272 (3)	Fe2—Cl1	2.4976 (11)
Mo1—N3	2.269 (3)	Fe2—P2	2.4019 (12)
Mo1—N5	2.265 (3)	Fe3—S2	2.2464 (11)
Fe1—Fe2	2.5896 (8)	Fe3—S3	2.2538 (11)
Fe1—Fe3	2.6085 (7)	Fe3—Cl1	2.4792 (11)
Fe1—S1	2.2573 (10)	Fe3—P3	2.3930 (10)
			
Fe1—Cl1—Fe3	63.64 (3)	Fe3—Cl1—Fe2	62.37 (3)

**Table 2:** Experimental details

Crystal data	
Chemical formula	[Fe_3_MoClS_3_(C_15_H_22_BN_6_)(C_3_H_9_P)_3_](C_24_H_20_B)
*M*_r_	1239.74
Crystal system, space group	Triclinic, *P* 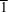
Temperature (K)	193
*a*, *b*, *c* (Å)	13.5563 (6), 14.6993 (6), 16.4158 (7)
α, β, γ (°)	76.650 (2), 86.558 (2), 89.408 (2)
*V* (Å^3^)	3177.0 (2)
*Z*	2
Radiation type	Ga *K*α, λ = 1.34138 Å
μ (mm^−1^)	6.17
Crystal size (mm)	0.04 × 0.03 × 0.02

Data collection	
Diffractometer	Bruker APEXII CCD
Absorption correction	Multi-scan (*SADABS*; Krause et al., 2015)
*T*_min_, *T*_max_	0.358, 0.750
No. of measured, independent and observed [*I* > 2σ(*I*)] reflections	24282, 10840, 9181
*R*_int_	0.059
(sin θ/λ)_max_ (Å^−1^)	0.596

Refinement	
*R*[*F*^2^ > 2σ(*F*^2^)], *wR*(*F*^2^), *S*	0.047, 0.130, 1.09
No. of reflections	10840
No. of parameters	622
No. of restraints	1
H-atom treatment	H-atom parameters constrained
Δρ_max_, Δρ_min_ (e Å^−3^)	1.38, −1.44

Computer programs: *APEX2* and *SAINT* (Bruker, 2015 ▸), *OLEX2.solve* (Bourhis *et al.*, 2015 ▸), *SHELXL2019/3* (Sheldrick, 2015 ▸) and *OLEX2* (Dolomanov *et al.*, 2009 ▸).

## supplementary crystallographic information

### µ3-Chlorido-tri-µ3-sulfido-tris(trimethylphosphine)[tris(3,5-dimethylpyrazol-1-yl)hydroborato]triiron(II)molybdenum(III) tetraphenylborate Crystal data

**Table:**

[Fe_3_MoClS_3_(C_15_H_22_BN_6_)(C_3_H_9_P)_3_](C_24_H_20_B)	*Z* = 2
*M**_r_* = 1239.74	*F*(000) = 1278
Triclinic, *P*1	*D*_x_ = 1.296 Mg m^−^^3^
*a* = 13.5563 (6) Å	Ga *K*α radiation, λ = 1.34138 Å
*b* = 14.6993 (6) Å	Cell parameters from 9880 reflections
*c* = 16.4158 (7) Å	θ = 3.8–53.0°
α = 76.650 (2)°	µ = 6.17 mm^−^^1^
β = 86.558 (2)°	*T* = 193 K
γ = 89.408 (2)°	Block, dark black
*V* = 3177.0 (2) Å^3^	0.04 × 0.03 × 0.02 mm

### µ3-Chlorido-tri-µ3-sulfido-tris(trimethylphosphine)[tris(3,5-dimethylpyrazol-1-yl)hydroborato]triiron(II)molybdenum(III) tetraphenylborate Data collection

**Table:**

Bruker APEXII CCD diffractometer	9181 reflections with *I* > 2σ(*I*)
φ and ω scans	*R*_int_ = 0.059
Absorption correction: multi-scan (SADABS; Krause et al., 2015)	θ_max_ = 53.1°, θ_min_ = 3.8°
*T*_min_ = 0.358, *T*_max_ = 0.750	*h* = −16→15
24282 measured reflections	*k* = −17→17
10840 independent reflections	*l* = −19→19

### µ3-Chlorido-tri-µ3-sulfido-tris(trimethylphosphine)[tris(3,5-dimethylpyrazol-1-yl)hydroborato]triiron(II)molybdenum(III) tetraphenylborate Refinement

**Table:**

Refinement on *F*^2^	1 restraint
Least-squares matrix: full	Hydrogen site location: mixed
*R*[*F*^2^ > 2σ(*F*^2^)] = 0.047	H atoms treated by a mixture of independent and constrained refinement
*wR*(*F*^2^) = 0.130	*w* = 1/[σ^2^(*F*_o_^2^) + (0.0629*P*)^2^ + 0.422*P*] where *P* = (*F*_o_^2^ + 2*F*_c_^2^)/3
*S* = 1.09	(Δ/σ)_max_ = 0.001
10840 reflections	Δρ_max_ = 1.38 e Å^−^^3^
622 parameters	Δρ_min_ = −1.44 e Å^−^^3^

### µ3-Chlorido-tri-µ3-sulfido-tris(trimethylphosphine)[tris(3,5-dimethylpyrazol-1-yl)hydroborato]triiron(II)molybdenum(III) tetraphenylborate Special details

**Table:**

Experimental. Single-crystal X-ray diffraction data for the title compound was collected at 193 K on a Bruker APEX II CCD diffractometer operating at 50 kV and 30 mA using Ga-Kα radiation (λ = 1.34138 Å). Crystal was mounted on a loop using Parabar 10312 oil for data collection. Data was collected with a series of *φ* and/or *ω* scans. Data was integrated using SAINT and scaled with either a numerical or multiscan absorption correction using SADABS. Structure was solved using SHELXT and refined by full-matrix least-squares on *F^2^* using the SHELXL and OLEX2 (Dolomanov *et al.*, 2009) programs. All non-hydrogen atoms were refined with anisotropic displacement parameters.
Geometry. All esds (except the esd in the dihedral angle between two l.s. planes) are estimated using the full covariance matrix. The cell esds are taken into account individually in the estimation of esds in distances, angles and torsion angles; correlations between esds in cell parameters are only used when they are defined by crystal symmetry. An approximate (isotropic) treatment of cell esds is used for estimating esds involving l.s. planes.

### µ3-Chlorido-tri-µ3-sulfido-tris(trimethylphosphine)[tris(3,5-dimethylpyrazol-1-yl)hydroborato]triiron(II)molybdenum(III) tetraphenylborate Fractional atomic coordinates and isotropic or equivalent isotropic displacement parameters (Å^2^)

**Table:**

	*x*	*y*	*z*	*U*_iso_*/*U*_eq_	
Mo1	0.75812 (2)	0.33475 (2)	0.48764 (2)	0.01710 (10)	
Fe1	0.70662 (4)	0.46086 (4)	0.57946 (3)	0.02321 (15)	
Fe2	0.85269 (4)	0.34927 (4)	0.62444 (3)	0.02376 (15)	
Fe3	0.67512 (4)	0.28355 (4)	0.64487 (3)	0.02361 (15)	
S1	0.85371 (7)	0.46348 (6)	0.50678 (6)	0.0225 (2)	
S2	0.80741 (7)	0.21129 (6)	0.60013 (6)	0.0240 (2)	
S3	0.59614 (6)	0.37183 (6)	0.53748 (6)	0.0242 (2)	
Cl1	0.72983 (8)	0.39173 (7)	0.72898 (6)	0.0340 (2)	
P3	0.59876 (8)	0.18825 (7)	0.77049 (6)	0.0289 (2)	
P2	0.98350 (8)	0.36536 (8)	0.71259 (6)	0.0331 (3)	
P1	0.65672 (8)	0.60423 (7)	0.61598 (7)	0.0299 (2)	
N1	0.7243 (2)	0.4251 (2)	0.36033 (18)	0.0217 (7)	
N3	0.8900 (2)	0.2929 (2)	0.41267 (18)	0.0229 (7)	
N2	0.7485 (2)	0.3916 (2)	0.28978 (18)	0.0239 (7)	
N5	0.6810 (2)	0.2256 (2)	0.43663 (19)	0.0234 (7)	
N4	0.8745 (2)	0.2695 (2)	0.33785 (18)	0.0248 (7)	
N6	0.6954 (2)	0.2275 (2)	0.35250 (18)	0.0251 (7)	
C18	0.7328 (3)	0.4581 (3)	0.2196 (2)	0.0275 (9)	
C16	0.6929 (3)	0.5134 (2)	0.3318 (2)	0.0228 (8)	
C17	0.6971 (3)	0.5355 (3)	0.2452 (2)	0.0283 (9)	
H17	0.678776	0.593265	0.210030	0.034*	
C15	0.6562 (3)	0.5760 (3)	0.3865 (2)	0.0299 (9)	
H15A	0.596567	0.548815	0.419544	0.045*	
H15B	0.640499	0.637455	0.351619	0.045*	
H15C	0.707287	0.583048	0.424417	0.045*	
B1	0.7747 (3)	0.2893 (3)	0.2976 (3)	0.0255 (10)	
H1	0.785 (3)	0.273 (2)	0.2336 (14)	0.031*	
C10	0.5741 (3)	0.1323 (3)	0.5572 (2)	0.0358 (10)	
H10A	0.629522	0.110497	0.592237	0.054*	
H10B	0.525283	0.081972	0.564336	0.054*	
H10C	0.543272	0.186343	0.573880	0.054*	
C36	0.2274 (3)	0.3095 (3)	0.0543 (2)	0.0308 (9)	
C26	0.2971 (3)	0.1698 (3)	−0.0194 (2)	0.0264 (9)	
C35	0.2453 (3)	0.3845 (3)	0.0898 (3)	0.0382 (10)	
H35	0.309644	0.392179	0.107096	0.046*	
C21	0.9848 (3)	0.2710 (2)	0.4281 (2)	0.0261 (9)	
C37	0.4233 (3)	0.2742 (3)	0.0303 (2)	0.0286 (9)	
C29	0.2878 (3)	0.0676 (3)	−0.1455 (3)	0.0449 (12)	
H29	0.283764	0.033111	−0.187455	0.054*	
C20	1.0343 (3)	0.2897 (3)	0.5013 (3)	0.0340 (10)	
H20A	1.037888	0.357338	0.496372	0.051*	
H20B	1.101196	0.263756	0.502483	0.051*	
H20C	0.996233	0.260359	0.553230	0.051*	
C30	0.2518 (4)	0.1568 (4)	−0.1580 (3)	0.0484 (12)	
H30	0.223187	0.184617	−0.209290	0.058*	
C25	0.2567 (3)	0.2064 (3)	−0.0965 (3)	0.0344 (10)	
H25	0.231511	0.268306	−0.106995	0.041*	
C44	0.3496 (3)	0.1731 (3)	0.2144 (3)	0.0395 (11)	
H44	0.401354	0.218100	0.204568	0.047*	
C13	0.6349 (3)	0.1650 (3)	0.3321 (3)	0.0326 (10)	
C12	0.5801 (3)	0.1221 (3)	0.4033 (3)	0.0364 (10)	
H12	0.530607	0.075581	0.407893	0.044*	
C19	0.7555 (4)	0.4439 (3)	0.1334 (2)	0.0449 (12)	
H19A	0.819033	0.411798	0.131531	0.067*	
H19B	0.759148	0.504729	0.093319	0.067*	
H19C	0.703346	0.405916	0.118810	0.067*	
C43	0.2978 (3)	0.1603 (3)	0.1466 (2)	0.0304 (9)	
C11	0.6112 (3)	0.1601 (2)	0.4670 (2)	0.0272 (9)	
C34	0.1720 (4)	0.4492 (3)	0.1010 (3)	0.0469 (13)	
H34	0.187326	0.499386	0.125644	0.056*	
C7	0.5014 (3)	0.2508 (3)	0.8140 (3)	0.0445 (12)	
H7A	0.527977	0.309105	0.823272	0.067*	
H7B	0.474850	0.212050	0.867485	0.067*	
H7C	0.448567	0.265218	0.774842	0.067*	
C42	0.5089 (3)	0.2205 (3)	0.0510 (3)	0.0379 (10)	
H42	0.500927	0.157968	0.082684	0.045*	
C48	0.2236 (3)	0.0928 (3)	0.1663 (3)	0.0397 (11)	
H48	0.186585	0.081303	0.122284	0.048*	
C22	1.0275 (3)	0.2339 (3)	0.3651 (3)	0.0319 (10)	
H22	1.093600	0.212318	0.361646	0.038*	
C38	0.4423 (3)	0.3640 (3)	−0.0191 (3)	0.0395 (11)	
H38	0.387910	0.403095	−0.037527	0.047*	
C23	0.9575 (3)	0.2334 (3)	0.3080 (2)	0.0313 (9)	
C5	1.1063 (3)	0.3283 (3)	0.6841 (3)	0.0458 (12)	
H5A	1.105196	0.261394	0.685091	0.069*	
H5B	1.152672	0.339834	0.724150	0.069*	
H5C	1.127315	0.363659	0.627569	0.069*	
C46	0.2527 (4)	0.0555 (3)	0.3127 (3)	0.0521 (14)	
H46	0.237499	0.020448	0.368276	0.063*	
C45	0.3272 (4)	0.1214 (3)	0.2964 (3)	0.0470 (13)	
H45	0.363596	0.131936	0.341103	0.056*	
C31	0.1309 (3)	0.3022 (3)	0.0314 (3)	0.0410 (11)	
H31	0.114542	0.251462	0.007861	0.049*	
C9	0.5436 (4)	0.0762 (3)	0.7708 (3)	0.0439 (12)	
H9A	0.490519	0.085518	0.731713	0.066*	
H9B	0.516325	0.047345	0.827433	0.066*	
H9C	0.594054	0.035191	0.753419	0.066*	
C27	0.3345 (3)	0.0789 (3)	−0.0103 (3)	0.0358 (10)	
H27	0.364280	0.050486	0.040173	0.043*	
C28	0.3297 (3)	0.0289 (3)	−0.0719 (3)	0.0431 (11)	
H28	0.355738	−0.032690	−0.062925	0.052*	
C41	0.6030 (3)	0.2538 (4)	0.0276 (3)	0.0460 (12)	
H41	0.657959	0.214632	0.043900	0.055*	
C2	0.7470 (3)	0.6363 (3)	0.6812 (3)	0.0404 (11)	
H2A	0.747406	0.589168	0.734218	0.061*	
H2B	0.730009	0.697328	0.692509	0.061*	
H2C	0.812662	0.639798	0.652091	0.061*	
C40	0.6184 (4)	0.3438 (4)	−0.0192 (3)	0.0505 (13)	
H40	0.683488	0.367230	−0.035074	0.061*	
C47	0.2012 (4)	0.0412 (3)	0.2477 (3)	0.0479 (13)	
H47	0.149695	−0.004100	0.258101	0.058*	
C1	0.6454 (4)	0.7099 (3)	0.5335 (3)	0.0451 (12)	
H1A	0.708555	0.723254	0.500797	0.068*	
H1B	0.627495	0.762500	0.558775	0.068*	
H1C	0.593949	0.700804	0.496639	0.068*	
B2	0.3121 (4)	0.2287 (3)	0.0521 (3)	0.0296 (10)	
C6	0.9605 (4)	0.3060 (4)	0.8217 (3)	0.0679 (17)	
H6A	0.901849	0.332658	0.845089	0.102*	
H6B	1.017736	0.313841	0.853137	0.102*	
H6C	0.949421	0.239225	0.825764	0.102*	
C33	0.0779 (4)	0.4403 (3)	0.0763 (3)	0.0496 (13)	
H33	0.028158	0.484428	0.082971	0.060*	
C8	0.6869 (4)	0.1586 (3)	0.8510 (3)	0.0476 (12)	
H8A	0.740033	0.120869	0.832726	0.071*	
H8B	0.653491	0.122857	0.903020	0.071*	
H8C	0.714761	0.216058	0.860983	0.071*	
C32	0.0576 (3)	0.3661 (3)	0.0417 (3)	0.0457 (12)	
H32	−0.007043	0.358644	0.024659	0.055*	
C4	1.0033 (3)	0.4859 (3)	0.7165 (3)	0.0445 (11)	
H4A	1.024670	0.522292	0.660416	0.067*	
H4B	1.054433	0.488800	0.755560	0.067*	
H4C	0.941595	0.511830	0.735514	0.067*	
C39	0.5378 (4)	0.3988 (3)	−0.0424 (3)	0.0512 (13)	
H39	0.547030	0.460942	−0.074687	0.061*	
C14	0.6316 (4)	0.1516 (3)	0.2442 (3)	0.0525 (13)	
H14A	0.613536	0.210649	0.206652	0.079*	
H14B	0.582378	0.103597	0.243080	0.079*	
H14C	0.696712	0.131774	0.225639	0.079*	
C24	0.9652 (4)	0.2031 (3)	0.2277 (3)	0.0502 (13)	
H24A	0.918605	0.151721	0.230683	0.075*	
H24B	1.032621	0.181815	0.217759	0.075*	
H24C	0.949363	0.255701	0.181759	0.075*	
C3	0.5411 (3)	0.6000 (4)	0.6782 (4)	0.0587 (14)	
H3A	0.486636	0.587980	0.645376	0.088*	
H3B	0.530334	0.660000	0.693784	0.088*	
H3C	0.543668	0.549894	0.729044	0.088*	

### µ3-Chlorido-tri-µ3-sulfido-tris(trimethylphosphine)[tris(3,5-dimethylpyrazol-1-yl)hydroborato]triiron(II)molybdenum(III) tetraphenylborate Atomic displacement parameters (Å^2^)

**Table:**

	*U*^11^	*U*^22^	*U*^33^	*U*^12^	*U*^13^	*U*^23^
Mo1	0.01712 (17)	0.01653 (16)	0.01822 (16)	−0.00150 (11)	−0.00160 (12)	−0.00500 (12)
Fe1	0.0257 (3)	0.0211 (3)	0.0245 (3)	−0.0017 (2)	−0.0001 (2)	−0.0091 (2)
Fe2	0.0246 (3)	0.0251 (3)	0.0218 (3)	−0.0043 (2)	−0.0056 (2)	−0.0045 (2)
Fe3	0.0266 (3)	0.0233 (3)	0.0205 (3)	−0.0052 (2)	0.0031 (2)	−0.0052 (2)
S1	0.0228 (5)	0.0204 (5)	0.0245 (5)	−0.0048 (4)	−0.0021 (4)	−0.0051 (4)
S2	0.0271 (5)	0.0192 (5)	0.0248 (5)	0.0007 (4)	−0.0014 (4)	−0.0036 (4)
S3	0.0176 (5)	0.0277 (5)	0.0288 (5)	−0.0008 (4)	0.0007 (4)	−0.0102 (4)
Cl1	0.0461 (6)	0.0325 (5)	0.0252 (5)	−0.0081 (4)	0.0012 (4)	−0.0108 (4)
P3	0.0349 (6)	0.0248 (5)	0.0255 (5)	−0.0012 (4)	0.0071 (4)	−0.0053 (4)
P2	0.0316 (6)	0.0377 (6)	0.0283 (5)	−0.0076 (5)	−0.0103 (5)	−0.0018 (5)
P1	0.0261 (5)	0.0279 (5)	0.0406 (6)	−0.0019 (4)	−0.0015 (5)	−0.0180 (5)
N1	0.0225 (16)	0.0199 (16)	0.0223 (16)	0.0014 (13)	−0.0027 (13)	−0.0038 (13)
N3	0.0248 (17)	0.0228 (16)	0.0216 (16)	−0.0007 (13)	−0.0001 (13)	−0.0066 (13)
N2	0.0314 (18)	0.0234 (16)	0.0186 (15)	0.0014 (14)	−0.0051 (13)	−0.0075 (13)
N5	0.0232 (17)	0.0234 (16)	0.0251 (16)	−0.0028 (13)	−0.0032 (13)	−0.0080 (13)
N4	0.0302 (18)	0.0246 (16)	0.0213 (16)	0.0038 (14)	0.0013 (14)	−0.0098 (13)
N6	0.0340 (19)	0.0219 (16)	0.0229 (16)	−0.0034 (14)	−0.0069 (14)	−0.0108 (13)
C18	0.031 (2)	0.031 (2)	0.0207 (19)	−0.0027 (17)	−0.0070 (17)	−0.0044 (16)
C16	0.0185 (19)	0.024 (2)	0.0261 (19)	−0.0011 (15)	−0.0023 (16)	−0.0067 (16)
C17	0.035 (2)	0.023 (2)	0.026 (2)	0.0037 (17)	−0.0069 (17)	−0.0009 (16)
C15	0.032 (2)	0.026 (2)	0.032 (2)	0.0099 (17)	−0.0014 (18)	−0.0071 (17)
B1	0.034 (3)	0.026 (2)	0.020 (2)	0.0041 (19)	−0.0037 (19)	−0.0104 (18)
C10	0.040 (2)	0.030 (2)	0.035 (2)	−0.0104 (19)	−0.0020 (19)	−0.0011 (18)
C36	0.041 (3)	0.026 (2)	0.0211 (19)	0.0058 (18)	0.0031 (18)	0.0010 (16)
C26	0.027 (2)	0.025 (2)	0.025 (2)	−0.0023 (16)	0.0015 (16)	−0.0020 (16)
C35	0.048 (3)	0.036 (2)	0.029 (2)	0.014 (2)	−0.001 (2)	−0.0054 (19)
C21	0.019 (2)	0.0223 (19)	0.034 (2)	0.0028 (16)	−0.0016 (17)	−0.0016 (17)
C37	0.034 (2)	0.028 (2)	0.027 (2)	0.0003 (18)	0.0001 (17)	−0.0113 (17)
C29	0.046 (3)	0.047 (3)	0.048 (3)	−0.002 (2)	−0.003 (2)	−0.024 (2)
C20	0.021 (2)	0.037 (2)	0.044 (3)	0.0021 (18)	−0.0001 (18)	−0.011 (2)
C30	0.052 (3)	0.059 (3)	0.039 (3)	0.000 (2)	−0.007 (2)	−0.019 (2)
C25	0.035 (2)	0.034 (2)	0.033 (2)	0.0030 (19)	0.0024 (19)	−0.0063 (19)
C44	0.054 (3)	0.032 (2)	0.035 (2)	0.018 (2)	−0.004 (2)	−0.0119 (19)
C13	0.043 (3)	0.024 (2)	0.036 (2)	−0.0004 (18)	−0.016 (2)	−0.0138 (18)
C12	0.042 (3)	0.029 (2)	0.041 (2)	−0.0143 (19)	−0.009 (2)	−0.0107 (19)
C19	0.074 (3)	0.040 (3)	0.021 (2)	0.007 (2)	−0.009 (2)	−0.0062 (19)
C43	0.035 (2)	0.029 (2)	0.024 (2)	0.0120 (18)	0.0016 (18)	−0.0011 (17)
C11	0.029 (2)	0.0196 (19)	0.033 (2)	−0.0021 (16)	−0.0056 (17)	−0.0060 (16)
C34	0.073 (4)	0.038 (3)	0.029 (2)	0.021 (2)	0.001 (2)	−0.007 (2)
C7	0.049 (3)	0.042 (3)	0.043 (3)	0.001 (2)	0.019 (2)	−0.017 (2)
C42	0.040 (3)	0.039 (2)	0.036 (2)	−0.001 (2)	−0.002 (2)	−0.011 (2)
C48	0.036 (3)	0.042 (3)	0.036 (2)	0.005 (2)	0.003 (2)	0.001 (2)
C22	0.024 (2)	0.027 (2)	0.046 (3)	0.0039 (17)	0.0095 (19)	−0.0140 (18)
C38	0.050 (3)	0.031 (2)	0.038 (2)	−0.004 (2)	0.008 (2)	−0.0124 (19)
C23	0.036 (2)	0.028 (2)	0.030 (2)	0.0027 (18)	0.0070 (19)	−0.0107 (17)
C5	0.036 (3)	0.046 (3)	0.059 (3)	0.004 (2)	−0.022 (2)	−0.014 (2)
C46	0.070 (4)	0.043 (3)	0.036 (3)	0.027 (3)	0.018 (3)	0.001 (2)
C45	0.075 (4)	0.042 (3)	0.025 (2)	0.026 (3)	−0.007 (2)	−0.010 (2)
C31	0.043 (3)	0.041 (3)	0.035 (2)	0.006 (2)	0.006 (2)	−0.005 (2)
C9	0.060 (3)	0.027 (2)	0.043 (3)	−0.009 (2)	0.019 (2)	−0.0101 (19)
C27	0.042 (3)	0.030 (2)	0.035 (2)	0.0039 (19)	−0.0011 (19)	−0.0055 (18)
C28	0.049 (3)	0.029 (2)	0.054 (3)	0.000 (2)	0.005 (2)	−0.016 (2)
C41	0.040 (3)	0.066 (3)	0.038 (3)	−0.002 (2)	−0.001 (2)	−0.024 (2)
C2	0.043 (3)	0.037 (2)	0.049 (3)	0.002 (2)	−0.014 (2)	−0.024 (2)
C40	0.045 (3)	0.070 (4)	0.044 (3)	−0.019 (3)	0.006 (2)	−0.031 (3)
C47	0.047 (3)	0.043 (3)	0.044 (3)	0.005 (2)	0.015 (2)	0.004 (2)
C1	0.053 (3)	0.033 (2)	0.054 (3)	0.002 (2)	−0.016 (2)	−0.014 (2)
B2	0.035 (3)	0.027 (2)	0.025 (2)	0.005 (2)	−0.001 (2)	−0.0020 (19)
C6	0.071 (4)	0.090 (4)	0.032 (3)	−0.024 (3)	−0.021 (3)	0.012 (3)
C33	0.061 (3)	0.048 (3)	0.035 (3)	0.033 (3)	0.006 (2)	−0.004 (2)
C8	0.063 (3)	0.040 (3)	0.036 (3)	0.005 (2)	−0.007 (2)	0.000 (2)
C32	0.040 (3)	0.048 (3)	0.043 (3)	0.021 (2)	0.003 (2)	−0.001 (2)
C4	0.045 (3)	0.048 (3)	0.046 (3)	−0.009 (2)	−0.004 (2)	−0.022 (2)
C39	0.071 (4)	0.040 (3)	0.044 (3)	−0.019 (3)	0.019 (3)	−0.018 (2)
C14	0.073 (4)	0.051 (3)	0.042 (3)	−0.013 (3)	−0.019 (3)	−0.024 (2)
C24	0.059 (3)	0.050 (3)	0.043 (3)	0.013 (2)	0.014 (2)	−0.020 (2)
C3	0.037 (3)	0.071 (4)	0.076 (4)	−0.002 (3)	0.008 (3)	−0.037 (3)

### µ3-Chlorido-tri-µ3-sulfido-tris(trimethylphosphine)[tris(3,5-dimethylpyrazol-1-yl)hydroborato]triiron(II)molybdenum(III) tetraphenylborate Geometric parameters (Å, º)

**Table:**

Mo1—Fe1	2.7080 (6)	C30—H30	0.9500
Mo1—Fe2	2.7028 (6)	C30—C25	1.381 (6)
Mo1—Fe3	2.6955 (6)	C25—H25	0.9500
Mo1—S1	2.3944 (9)	C44—H44	0.9500
Mo1—S2	2.3928 (9)	C44—C43	1.400 (6)
Mo1—S3	2.3986 (9)	C44—C45	1.402 (6)
Mo1—N1	2.272 (3)	C13—C12	1.372 (6)
Mo1—N3	2.269 (3)	C13—C14	1.504 (5)
Mo1—N5	2.265 (3)	C12—H12	0.9500
Fe1—Fe2	2.5896 (8)	C12—C11	1.383 (5)
Fe1—Fe3	2.6085 (7)	C19—H19A	0.9800
Fe1—S1	2.2573 (10)	C19—H19B	0.9800
Fe1—S3	2.2387 (11)	C19—H19C	0.9800
Fe1—Cl1	2.4678 (11)	C43—C48	1.391 (6)
Fe1—P1	2.4006 (11)	C43—B2	1.644 (5)
Fe2—Fe3	2.5772 (8)	C34—H34	0.9500
Fe2—S1	2.2470 (10)	C34—C33	1.378 (7)
Fe2—S2	2.2536 (10)	C7—H7A	0.9800
Fe2—Cl1	2.4976 (11)	C7—H7B	0.9800
Fe2—P2	2.4019 (12)	C7—H7C	0.9800
Fe3—S2	2.2464 (11)	C42—H42	0.9500
Fe3—S3	2.2538 (11)	C42—C41	1.372 (6)
Fe3—Cl1	2.4792 (11)	C48—H48	0.9500
Fe3—P3	2.3930 (10)	C48—C47	1.394 (6)
P3—C7	1.806 (4)	C22—H22	0.9500
P3—C9	1.815 (4)	C22—C23	1.374 (6)
P3—C8	1.810 (5)	C38—H38	0.9500
P2—C5	1.814 (5)	C38—C39	1.395 (6)
P2—C6	1.812 (5)	C23—C24	1.483 (5)
P2—C4	1.811 (4)	C5—H5A	0.9800
P1—C2	1.808 (4)	C5—H5B	0.9800
P1—C1	1.821 (4)	C5—H5C	0.9800
P1—C3	1.813 (5)	C46—H46	0.9500
N1—N2	1.381 (4)	C46—C45	1.377 (7)
N1—C16	1.348 (4)	C46—C47	1.367 (7)
N3—N4	1.378 (4)	C45—H45	0.9500
N3—C21	1.347 (5)	C31—H31	0.9500
N2—C18	1.354 (4)	C31—C32	1.390 (6)
N2—B1	1.520 (5)	C9—H9A	0.9800
N5—N6	1.377 (4)	C9—H9B	0.9800
N5—C11	1.346 (5)	C9—H9C	0.9800
N4—B1	1.537 (5)	C27—H27	0.9500
N4—C23	1.352 (5)	C27—C28	1.386 (6)
N6—B1	1.523 (5)	C28—H28	0.9500
N6—C13	1.349 (5)	C41—H41	0.9500
C18—C17	1.376 (5)	C41—C40	1.378 (7)
C18—C19	1.490 (5)	C2—H2A	0.9800
C16—C17	1.381 (5)	C2—H2B	0.9800
C16—C15	1.490 (5)	C2—H2C	0.9800
C17—H17	0.9500	C40—H40	0.9500
C15—H15A	0.9800	C40—C39	1.372 (7)
C15—H15B	0.9800	C47—H47	0.9500
C15—H15C	0.9800	C1—H1A	0.9800
B1—H1	1.128 (18)	C1—H1B	0.9800
C10—H10A	0.9800	C1—H1C	0.9800
C10—H10B	0.9800	C6—H6A	0.9800
C10—H10C	0.9800	C6—H6B	0.9800
C10—C11	1.498 (5)	C6—H6C	0.9800
C36—C35	1.389 (6)	C33—H33	0.9500
C36—C31	1.396 (6)	C33—C32	1.378 (7)
C36—B2	1.648 (6)	C8—H8A	0.9800
C26—C25	1.397 (6)	C8—H8B	0.9800
C26—C27	1.402 (5)	C8—H8C	0.9800
C26—B2	1.633 (6)	C32—H32	0.9500
C35—H35	0.9500	C4—H4A	0.9800
C35—C34	1.401 (6)	C4—H4B	0.9800
C21—C20	1.492 (5)	C4—H4C	0.9800
C21—C22	1.371 (5)	C39—H39	0.9500
C37—C42	1.410 (6)	C14—H14A	0.9800
C37—C38	1.399 (6)	C14—H14B	0.9800
C37—B2	1.641 (6)	C14—H14C	0.9800
C29—H29	0.9500	C24—H24A	0.9800
C29—C30	1.369 (6)	C24—H24B	0.9800
C29—C28	1.365 (6)	C24—H24C	0.9800
C20—H20A	0.9800	C3—H3A	0.9800
C20—H20B	0.9800	C3—H3B	0.9800
C20—H20C	0.9800	C3—H3C	0.9800
			
Fe2—Mo1—Fe1	57.188 (18)	C35—C36—C31	115.3 (4)
Fe3—Mo1—Fe1	57.729 (17)	C35—C36—B2	120.8 (4)
Fe3—Mo1—Fe2	57.032 (17)	C31—C36—B2	123.4 (4)
S1—Mo1—Fe1	52.07 (3)	C25—C26—C27	114.4 (4)
S1—Mo1—Fe2	51.88 (2)	C25—C26—B2	124.6 (4)
S1—Mo1—Fe3	97.57 (3)	C27—C26—B2	120.8 (3)
S1—Mo1—S3	101.79 (3)	C36—C35—H35	118.7
S2—Mo1—Fe1	97.80 (3)	C36—C35—C34	122.6 (5)
S2—Mo1—Fe2	52.06 (3)	C34—C35—H35	118.7
S2—Mo1—Fe3	51.98 (3)	N3—C21—C20	123.4 (3)
S2—Mo1—S1	101.76 (3)	N3—C21—C22	109.8 (4)
S2—Mo1—S3	102.29 (3)	C22—C21—C20	126.7 (3)
S3—Mo1—Fe1	51.57 (3)	C42—C37—B2	121.8 (3)
S3—Mo1—Fe2	96.81 (3)	C38—C37—C42	114.0 (4)
S3—Mo1—Fe3	52.12 (3)	C38—C37—B2	123.7 (4)
N1—Mo1—Fe1	97.21 (8)	C30—C29—H29	120.4
N1—Mo1—Fe2	138.74 (8)	C28—C29—H29	120.4
N1—Mo1—Fe3	139.62 (8)	C28—C29—C30	119.3 (4)
N1—Mo1—S1	87.00 (8)	C21—C20—H20A	109.5
N1—Mo1—S2	164.98 (8)	C21—C20—H20B	109.5
N1—Mo1—S3	87.60 (8)	C21—C20—H20C	109.5
N3—Mo1—Fe1	140.34 (8)	H20A—C20—H20B	109.5
N3—Mo1—Fe2	97.88 (8)	H20A—C20—H20C	109.5
N3—Mo1—Fe3	137.69 (7)	H20B—C20—H20C	109.5
N3—Mo1—S1	88.43 (8)	C29—C30—H30	119.9
N3—Mo1—S2	85.75 (8)	C29—C30—C25	120.2 (4)
N3—Mo1—S3	165.24 (8)	C25—C30—H30	119.9
N3—Mo1—N1	82.28 (10)	C26—C25—H25	118.5
N5—Mo1—Fe1	137.10 (8)	C30—C25—C26	123.0 (4)
N5—Mo1—Fe2	139.65 (8)	C30—C25—H25	118.5
N5—Mo1—Fe3	96.27 (8)	C43—C44—H44	119.1
N5—Mo1—S1	166.08 (8)	C43—C44—C45	121.9 (5)
N5—Mo1—S2	87.96 (8)	C45—C44—H44	119.1
N5—Mo1—S3	85.61 (8)	N6—C13—C12	107.9 (3)
N5—Mo1—N1	81.49 (11)	N6—C13—C14	122.7 (4)
N5—Mo1—N3	82.30 (11)	C12—C13—C14	129.4 (4)
Fe2—Fe1—Mo1	61.306 (18)	C13—C12—H12	126.9
Fe2—Fe1—Fe3	59.44 (2)	C13—C12—C11	106.3 (3)
Fe3—Fe1—Mo1	60.896 (18)	C11—C12—H12	126.9
S1—Fe1—Mo1	56.79 (3)	C18—C19—H19A	109.5
S1—Fe1—Fe2	54.72 (3)	C18—C19—H19B	109.5
S1—Fe1—Fe3	103.75 (3)	C18—C19—H19C	109.5
S1—Fe1—Cl1	108.72 (4)	H19A—C19—H19B	109.5
S1—Fe1—P1	116.23 (4)	H19A—C19—H19C	109.5
S3—Fe1—Mo1	57.07 (3)	H19B—C19—H19C	109.5
S3—Fe1—Fe2	104.38 (3)	C44—C43—B2	123.1 (4)
S3—Fe1—Fe3	54.77 (3)	C48—C43—C44	115.5 (4)
S3—Fe1—S1	111.63 (4)	C48—C43—B2	121.0 (4)
S3—Fe1—Cl1	107.21 (4)	N5—C11—C10	124.5 (3)
S3—Fe1—P1	120.45 (4)	N5—C11—C12	110.1 (4)
Cl1—Fe1—Mo1	109.51 (3)	C12—C11—C10	125.5 (4)
Cl1—Fe1—Fe2	59.13 (3)	C35—C34—H34	119.9
Cl1—Fe1—Fe3	58.39 (3)	C33—C34—C35	120.2 (5)
P1—Fe1—Mo1	161.31 (4)	C33—C34—H34	119.9
P1—Fe1—Fe2	131.57 (4)	P3—C7—H7A	109.5
P1—Fe1—Fe3	135.27 (4)	P3—C7—H7B	109.5
P1—Fe1—Cl1	89.07 (4)	P3—C7—H7C	109.5
Fe1—Fe2—Mo1	61.506 (18)	H7A—C7—H7B	109.5
Fe3—Fe2—Mo1	61.341 (18)	H7A—C7—H7C	109.5
Fe3—Fe2—Fe1	60.64 (2)	H7B—C7—H7C	109.5
S1—Fe2—Mo1	56.97 (3)	C37—C42—H42	118.3
S1—Fe2—Fe1	55.09 (3)	C41—C42—C37	123.5 (4)
S1—Fe2—Fe3	105.06 (3)	C41—C42—H42	118.3
S1—Fe2—S2	111.22 (4)	C43—C48—H48	118.6
S1—Fe2—Cl1	108.03 (4)	C43—C48—C47	122.8 (4)
S1—Fe2—P2	112.80 (4)	C47—C48—H48	118.6
S2—Fe2—Mo1	56.87 (3)	C21—C22—H22	126.1
S2—Fe2—Fe1	105.06 (3)	C21—C22—C23	107.7 (3)
S2—Fe2—Fe3	54.93 (3)	C23—C22—H22	126.1
S2—Fe2—Cl1	108.45 (4)	C37—C38—H38	118.6
S2—Fe2—P2	123.40 (4)	C39—C38—C37	122.8 (4)
Cl1—Fe2—Mo1	108.77 (3)	C39—C38—H38	118.6
Cl1—Fe2—Fe1	58.00 (3)	N4—C23—C22	106.3 (3)
Cl1—Fe2—Fe3	58.46 (3)	N4—C23—C24	123.7 (4)
P2—Fe2—Mo1	160.64 (4)	C22—C23—C24	130.0 (4)
P2—Fe2—Fe1	128.95 (4)	P2—C5—H5A	109.5
P2—Fe2—Fe3	136.81 (4)	P2—C5—H5B	109.5
P2—Fe2—Cl1	89.77 (4)	P2—C5—H5C	109.5
Fe1—Fe3—Mo1	61.375 (17)	H5A—C5—H5B	109.5
Fe2—Fe3—Mo1	61.627 (18)	H5A—C5—H5C	109.5
Fe2—Fe3—Fe1	59.91 (2)	H5B—C5—H5C	109.5
S2—Fe3—Mo1	57.05 (3)	C45—C46—H46	120.5
S2—Fe3—Fe1	104.67 (3)	C47—C46—H46	120.5
S2—Fe3—Fe2	55.19 (3)	C47—C46—C45	119.0 (4)
S2—Fe3—S3	112.02 (4)	C44—C45—H45	119.8
S2—Fe3—Cl1	109.34 (4)	C46—C45—C44	120.5 (5)
S2—Fe3—P3	110.68 (4)	C46—C45—H45	119.8
S3—Fe3—Mo1	57.14 (3)	C36—C31—H31	118.6
S3—Fe3—Fe1	54.24 (3)	C32—C31—C36	122.8 (5)
S3—Fe3—Fe2	104.33 (3)	C32—C31—H31	118.6
S3—Fe3—Cl1	106.35 (4)	P3—C9—H9A	109.5
S3—Fe3—P3	126.11 (4)	P3—C9—H9B	109.5
Cl1—Fe3—Mo1	109.56 (3)	P3—C9—H9C	109.5
Cl1—Fe3—Fe1	57.97 (3)	H9A—C9—H9B	109.5
Cl1—Fe3—Fe2	59.16 (3)	H9A—C9—H9C	109.5
P3—Fe3—Mo1	160.31 (4)	H9B—C9—H9C	109.5
P3—Fe3—Fe1	137.93 (4)	C26—C27—H27	118.6
P3—Fe3—Fe2	126.80 (4)	C28—C27—C26	122.8 (4)
P3—Fe3—Cl1	88.64 (4)	C28—C27—H27	118.6
Fe1—S1—Mo1	71.13 (3)	C29—C28—C27	120.2 (4)
Fe2—S1—Mo1	71.15 (3)	C29—C28—H28	119.9
Fe2—S1—Fe1	70.19 (3)	C27—C28—H28	119.9
Fe2—S2—Mo1	71.07 (3)	C42—C41—H41	119.8
Fe3—S2—Mo1	70.97 (3)	C42—C41—C40	120.5 (5)
Fe3—S2—Fe2	69.88 (3)	C40—C41—H41	119.8
Fe1—S3—Mo1	71.36 (3)	P1—C2—H2A	109.5
Fe1—S3—Fe3	70.99 (3)	P1—C2—H2B	109.5
Fe3—S3—Mo1	70.74 (3)	P1—C2—H2C	109.5
Fe1—Cl1—Fe2	62.87 (3)	H2A—C2—H2B	109.5
Fe1—Cl1—Fe3	63.64 (3)	H2A—C2—H2C	109.5
Fe3—Cl1—Fe2	62.37 (3)	H2B—C2—H2C	109.5
C7—P3—Fe3	111.14 (15)	C41—C40—H40	120.7
C7—P3—C9	104.3 (2)	C39—C40—C41	118.6 (5)
C7—P3—C8	105.2 (2)	C39—C40—H40	120.7
C9—P3—Fe3	121.07 (14)	C48—C47—H47	119.8
C8—P3—Fe3	110.52 (16)	C46—C47—C48	120.4 (5)
C8—P3—C9	103.2 (2)	C46—C47—H47	119.8
C5—P2—Fe2	117.72 (15)	P1—C1—H1A	109.5
C6—P2—Fe2	114.20 (17)	P1—C1—H1B	109.5
C6—P2—C5	104.8 (3)	P1—C1—H1C	109.5
C4—P2—Fe2	112.24 (16)	H1A—C1—H1B	109.5
C4—P2—C5	102.6 (2)	H1A—C1—H1C	109.5
C4—P2—C6	103.7 (2)	H1B—C1—H1C	109.5
C2—P1—Fe1	109.05 (14)	C26—B2—C36	113.6 (3)
C2—P1—C1	103.3 (2)	C26—B2—C37	105.0 (3)
C2—P1—C3	104.0 (2)	C26—B2—C43	110.7 (3)
C1—P1—Fe1	119.57 (16)	C37—B2—C36	111.7 (3)
C3—P1—Fe1	115.89 (18)	C37—B2—C43	113.4 (3)
C3—P1—C1	103.3 (2)	C43—B2—C36	102.7 (3)
N2—N1—Mo1	118.5 (2)	P2—C6—H6A	109.5
C16—N1—Mo1	135.4 (2)	P2—C6—H6B	109.5
C16—N1—N2	105.7 (3)	P2—C6—H6C	109.5
N4—N3—Mo1	118.7 (2)	H6A—C6—H6B	109.5
C21—N3—Mo1	135.0 (3)	H6A—C6—H6C	109.5
C21—N3—N4	105.6 (3)	H6B—C6—H6C	109.5
N1—N2—B1	120.6 (3)	C34—C33—H33	120.7
C18—N2—N1	110.3 (3)	C34—C33—C32	118.6 (4)
C18—N2—B1	128.6 (3)	C32—C33—H33	120.7
N6—N5—Mo1	118.5 (2)	P3—C8—H8A	109.5
C11—N5—Mo1	135.1 (3)	P3—C8—H8B	109.5
C11—N5—N6	106.0 (3)	P3—C8—H8C	109.5
N3—N4—B1	120.6 (3)	H8A—C8—H8B	109.5
C23—N4—N3	110.7 (3)	H8A—C8—H8C	109.5
C23—N4—B1	128.7 (3)	H8B—C8—H8C	109.5
N5—N6—B1	121.2 (3)	C31—C32—H32	119.8
C13—N6—N5	109.8 (3)	C33—C32—C31	120.5 (5)
C13—N6—B1	128.8 (3)	C33—C32—H32	119.8
N2—C18—C17	106.9 (3)	P2—C4—H4A	109.5
N2—C18—C19	122.9 (4)	P2—C4—H4B	109.5
C17—C18—C19	130.1 (4)	P2—C4—H4C	109.5
N1—C16—C17	110.0 (3)	H4A—C4—H4B	109.5
N1—C16—C15	124.5 (3)	H4A—C4—H4C	109.5
C17—C16—C15	125.5 (3)	H4B—C4—H4C	109.5
C18—C17—C16	107.0 (3)	C38—C39—H39	119.7
C18—C17—H17	126.5	C40—C39—C38	120.5 (4)
C16—C17—H17	126.5	C40—C39—H39	119.7
C16—C15—H15A	109.5	C13—C14—H14A	109.5
C16—C15—H15B	109.5	C13—C14—H14B	109.5
C16—C15—H15C	109.5	C13—C14—H14C	109.5
H15A—C15—H15B	109.5	H14A—C14—H14B	109.5
H15A—C15—H15C	109.5	H14A—C14—H14C	109.5
H15B—C15—H15C	109.5	H14B—C14—H14C	109.5
N2—B1—N4	109.2 (3)	C23—C24—H24A	109.5
N2—B1—N6	109.8 (3)	C23—C24—H24B	109.5
N2—B1—H1	110.6 (19)	C23—C24—H24C	109.5
N4—B1—H1	106.0 (19)	H24A—C24—H24B	109.5
N6—B1—N4	109.3 (3)	H24A—C24—H24C	109.5
N6—B1—H1	112.0 (19)	H24B—C24—H24C	109.5
H10A—C10—H10B	109.5	P1—C3—H3A	109.5
H10A—C10—H10C	109.5	P1—C3—H3B	109.5
H10B—C10—H10C	109.5	P1—C3—H3C	109.5
C11—C10—H10A	109.5	H3A—C3—H3B	109.5
C11—C10—H10B	109.5	H3A—C3—H3C	109.5
C11—C10—H10C	109.5	H3B—C3—H3C	109.5
			
Mo1—N1—N2—C18	−173.7 (2)	C37—C38—C39—C40	1.9 (7)
Mo1—N1—N2—B1	13.9 (4)	C29—C30—C25—C26	−0.4 (7)
Mo1—N1—C16—C17	172.4 (3)	C20—C21—C22—C23	−176.6 (4)
Mo1—N1—C16—C15	−9.3 (5)	C30—C29—C28—C27	0.8 (7)
Mo1—N3—N4—B1	11.7 (4)	C25—C26—C27—C28	−1.4 (6)
Mo1—N3—N4—C23	−170.9 (2)	C25—C26—B2—C36	31.9 (5)
Mo1—N3—C21—C20	−14.1 (6)	C25—C26—B2—C37	−90.5 (4)
Mo1—N3—C21—C22	168.6 (3)	C25—C26—B2—C43	146.8 (4)
Mo1—N5—N6—B1	10.9 (4)	C44—C43—C48—C47	0.3 (6)
Mo1—N5—N6—C13	−172.7 (2)	C44—C43—B2—C36	−90.8 (4)
Mo1—N5—C11—C10	−10.3 (6)	C44—C43—B2—C26	147.6 (4)
Mo1—N5—C11—C12	170.4 (3)	C44—C43—B2—C37	29.9 (5)
N1—N2—C18—C17	−0.5 (4)	C13—N6—B1—N2	117.6 (4)
N1—N2—C18—C19	177.7 (4)	C13—N6—B1—N4	−122.7 (4)
N1—N2—B1—N4	−69.0 (4)	C13—C12—C11—N5	1.3 (5)
N1—N2—B1—N6	50.8 (4)	C13—C12—C11—C10	−178.0 (4)
N1—C16—C17—C18	−0.2 (4)	C19—C18—C17—C16	−177.6 (4)
N3—N4—B1—N2	53.0 (4)	C43—C44—C45—C46	0.2 (7)
N3—N4—B1—N6	−67.2 (4)	C43—C48—C47—C46	−0.1 (7)
N3—N4—C23—C22	0.0 (4)	C11—N5—N6—B1	−176.0 (3)
N3—N4—C23—C24	−178.8 (4)	C11—N5—N6—C13	0.5 (4)
N3—C21—C22—C23	0.6 (4)	C34—C33—C32—C31	−0.5 (7)
N2—N1—C16—C17	−0.1 (4)	C42—C37—C38—C39	−2.9 (6)
N2—N1—C16—C15	178.2 (3)	C42—C37—B2—C36	159.1 (4)
N2—C18—C17—C16	0.4 (4)	C42—C37—B2—C26	−77.3 (4)
N5—N6—B1—N2	−66.6 (4)	C42—C37—B2—C43	43.7 (5)
N5—N6—B1—N4	53.1 (4)	C42—C41—C40—C39	−0.5 (7)
N5—N6—C13—C12	0.3 (4)	C48—C43—B2—C36	81.0 (5)
N5—N6—C13—C14	178.8 (4)	C48—C43—B2—C26	−40.7 (5)
N4—N3—C21—C20	176.7 (3)	C48—C43—B2—C37	−158.3 (4)
N4—N3—C21—C22	−0.6 (4)	C38—C37—C42—C41	2.3 (6)
N6—N5—C11—C10	178.2 (3)	C38—C37—B2—C36	−29.7 (5)
N6—N5—C11—C12	−1.1 (4)	C38—C37—B2—C26	93.9 (4)
N6—C13—C12—C11	−1.0 (5)	C38—C37—B2—C43	−145.1 (4)
C18—N2—B1—N4	120.1 (4)	C23—N4—B1—N2	−123.9 (4)
C18—N2—B1—N6	−120.1 (4)	C23—N4—B1—N6	115.9 (4)
C16—N1—N2—C18	0.3 (4)	C45—C44—C43—C48	−0.4 (6)
C16—N1—N2—B1	−172.1 (3)	C45—C44—C43—B2	171.8 (4)
C15—C16—C17—C18	−178.4 (4)	C45—C46—C47—C48	−0.1 (7)
B1—N2—C18—C17	171.2 (4)	C31—C36—C35—C34	−0.7 (6)
B1—N2—C18—C19	−10.6 (6)	C31—C36—B2—C26	29.6 (5)
B1—N4—C23—C22	177.1 (4)	C31—C36—B2—C37	148.2 (4)
B1—N4—C23—C24	−1.7 (6)	C31—C36—B2—C43	−90.0 (4)
B1—N6—C13—C12	176.4 (4)	C27—C26—C25—C30	1.4 (6)
B1—N6—C13—C14	−5.1 (6)	C27—C26—B2—C36	−154.8 (3)
C36—C35—C34—C33	−0.2 (7)	C27—C26—B2—C37	82.8 (4)
C36—C31—C32—C33	−0.6 (7)	C27—C26—B2—C43	−39.9 (5)
C26—C27—C28—C29	0.3 (7)	C28—C29—C30—C25	−0.8 (7)
C35—C36—C31—C32	1.1 (6)	C41—C40—C39—C38	−0.1 (7)
C35—C36—B2—C26	−158.6 (3)	C47—C46—C45—C44	0.0 (7)
C35—C36—B2—C37	−40.1 (5)	B2—C36—C35—C34	−173.1 (4)
C35—C36—B2—C43	81.7 (4)	B2—C36—C31—C32	173.3 (4)
C35—C34—C33—C32	0.8 (7)	B2—C26—C25—C30	175.1 (4)
C21—N3—N4—B1	−177.0 (3)	B2—C26—C27—C28	−175.3 (4)
C21—N3—N4—C23	0.4 (4)	B2—C37—C42—C41	174.3 (4)
C21—C22—C23—N4	−0.4 (4)	B2—C37—C38—C39	−174.7 (4)
C21—C22—C23—C24	178.3 (4)	B2—C43—C48—C47	−172.0 (4)
C37—C42—C41—C40	−0.7 (7)	C14—C13—C12—C11	−179.4 (4)

### Selected geometric parameters (Å,°).

**Table:**

Mo1-Fe1	2.7080 (6)	Fe1-S1	2.2573 (10)
Mo1-Fe2	2.7028 (6)	Fe1-S3	2.2387 (11)
Mo1-Fe3	2.6955 (6)	Fe2-S1	2.2471 (10)
Mo1-S1	2.3944 (9)	Fe2-S2	2.2536 (10)
Mo1-S2	2.3928 (9)	Fe3-S2	2.2464 (11)
Mo1-S3	2.3987 (9)	Fe3-S3	2.2538 (11)
Fe1-Fe2	2.5896 (8)	Fe1-Cl1	2.4677 (11)
Fe1-Fe3	2.6085 (7)	Fe2-Cl1	2.4974 (11)
Fe2-Fe3	2.5772 (8)	Fe3-Cl1	2.4791 (11)
Fe1-P1	2.4006 (11)	Fe2-P2	2.4019 (12)
Fe3-P3	2.3930 (11)	Fe1-Cl1-Fe2	62.87 (3)
Fe3-Cl1-Fe2	62.38 (3)	Fe1-Cl1-Fe3	63.65 (3)
